# The Challenges of Cardiac Resynchronization Therapy in Patients with Dilated Cardiomyopathy and Persistent Left Superior Vena Cava: A Case Report and Concise Literature Review

**DOI:** 10.3390/biomedicines11041205

**Published:** 2023-04-18

**Authors:** Silvius Alexandru Pescariu, Raluca Șoșdean, Monica Nicoleta Mircea, Adina Ionac, Sorin Pescariu

**Affiliations:** 1 Department VI—Cardiology, “Victor Babeș” University of Medicine and Pharmacy of Timișoara, E. Murgu Sq. No. 2, 300041 Timișoara, Romania; pescariu.alexandru@umft.ro (S.A.P.); adina.ionac@gmail.com (A.I.); sorinpescariu@yahoo.com (S.P.); 2 Research Center of the Institute for Cardiovascular Diseases, “Victor Babeș” University of Medicine and Pharmacy of Timișoara, G. Adam St. No. 13A, 300310 Timișoara, Romania; mircea.monica@yahoo.ro

**Keywords:** heart failure with reduced ejection fraction, dilated cardiomyopathy, cardiac resynchronization therapy, persistent left superior vena cava

## Abstract

Heart failure with reduced ejection fraction (HFrEF) is a chronic and debilitating disease, which requires extensive diagnostic and treatment resources in order to achieve an acceptable quality of life for the patient. While optimal medical treatment remains at the core of the disease’s management, interventional cardiology also plays a very important role. However, in very rare situations, interventionists might find cases especially challenging due to the presence of venous anomalies, such as persistent left superior vena cava (PLSVC), anomalies that may go undiscovered during the patient’s lifetime until venous cannulation is necessary. While these types of malformations also pose challenges in regards to standard pacemaker implantation, cardiac resynchronization (CRT) devices pose several additional challenges due to the complexity of the device and the necessity of finding an optimal position for the coronary sinus (CS) lead. We present the case of a 55-year-old male patient with advanced heart failure due to dilated cardiomyopathy (DCM) and LBBB who was a candidate for CRT-D therapy, describing the investigations that led to the discovery of the PLSVC as well as the technique and results of the intervention, while comparing our case to similar cases found in recent literature.

## 1. Introduction

Heart failure with reduced ejection fraction (HFrEF) in the setting of dilated cardiomyopathy (DCM) with left bundle branch block (LBB) is a chronic disease that necessitates extensive diagnostic and treatment resources in order to achieve an acceptable quality of life for the patient. While optimal medical treatment remains at the core of the disease’s management, interventional cardiology also plays a very important role. Current treatment guidelines recommend cardiac resynchronization therapy (CRT) with pacemakers (CRT-P) and/or implantable cardiac defibrillators (ICD-CRT-D) for selected patients who suffer from HFrEF and LBBB [1]. While our clinic has significant experience in treating such patients, with most of them receiving CRT-P and/or CRT-D devices without encountering significant problems, some of these patients may exhibit exceptional anatomical abnormalities that stand in the way of achieving optimal interventional treatment. On the other hand, higher degrees of fibrosis and worse speckle tracking longitudinal strain results may also lead to suboptimal resynchronization parameters [2]. Venous anomalies such as persistent left superior vena cava (PLSVC) have been proved to be a challenge for implantologists regarding the insertion of permanent pacing leads [3], and the implantation of CRT devices raises additional problems as it also requires the positioning of a coronary sinus (CS) lead. Compared to CRT-P, CRT-D devices may induce supplemental technical difficulties as they require a thicker defibrillation lead positioned in the right ventricle.

PLSVC is considered to be the most frequently described venous congenital anomaly, with a prevalence reaching 0.5% within the general population [4]. During the embryonic stages of development, venous drainage of the upper half of the body is achieved via the routes of the left and right superior cardinal veins. While in normal individuals the left cardinal vein devolves into the so called “Ligament of Marshall”, in some individuals its existence continues, resulting in a left-sided vein, which drains into the coronary sinus [5]. Often, the venous abnormality consists of the presence of a left as well as a right superior vena cava (SVC), which may or may not be joined by an innominate vein [6]. The presence of PLSVC is most frequently diagnosed accidentally and generally does not raise particular concerns within the healthy adult population [7].

Although it is regarded as a benign malformation for most individuals, PLSVC can be troublesome in patients requiring an implantable cardiac device, as these devices require intracavitary leads, which are placed via the routes of the superior vena cava. These situations can be especially challenging when faced with patients in need of CRT-D therapy.

## 2. Case Report

**Figure 1:**
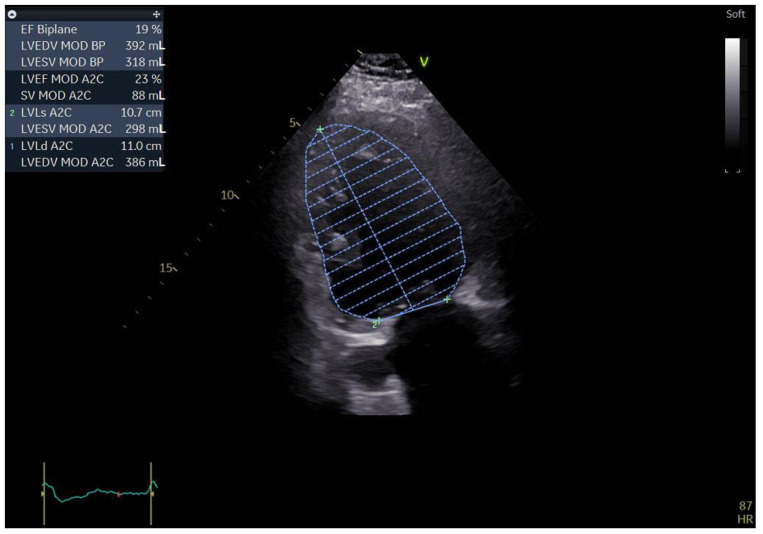
Two-dimensional echocardiography: apical two chamber view showing a severely dilated left ventricle along with a severely impaired LVEF; the top left of the picture showing the aforementioned measurements (biplane results).

**Figure 2:**
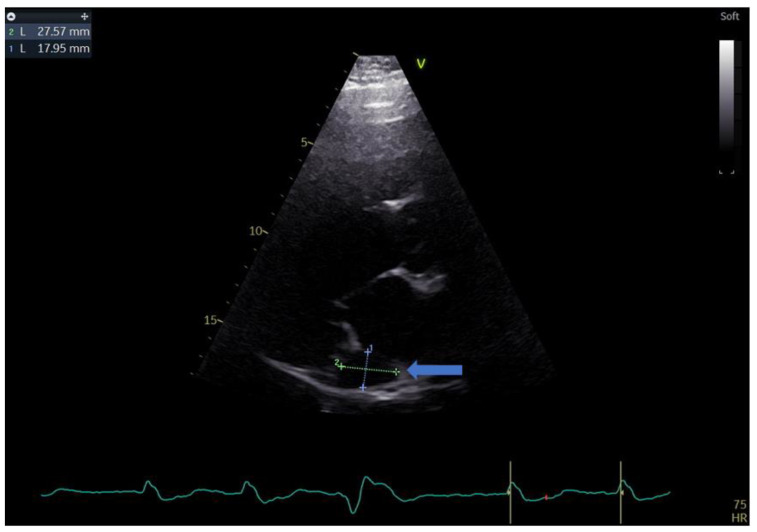
Parasternal long-axis view showing the measurements of the coronary sinus (blue arrow).

A 55-year-old male with a history of coronary artery disease was admitted to the hospital for shortness of breath, peripheral edema of the lower extremities, exercise intolerance and symptoms of heart failure, which could be classified as class III within the classification of the New York Heart Association (NYHA). The standard 12 lead ECG revealed a heart in atrial fibrillation (AFib) and a LBBB with a QRS complex measuring 140 ms and a heart rate of 90 b/min. Echocardiography performed upon admission revealed an extremely dilated left ventricle with a severely reduced ejection fraction. The patient had an end-diastolic volume of 392 mL with an and-systolic volume of 318 mL, resulting in a biplane left ventricular ejection fraction (LVEF) of 19%. During the echo exam, we came across a rather unusual finding in the form of a significantly dilated coronary sinus, measuring 27 by 18 mm in the parasternal long-axis (PLAX) view (Figure 1 and Figure 2).

**Figure 3:**
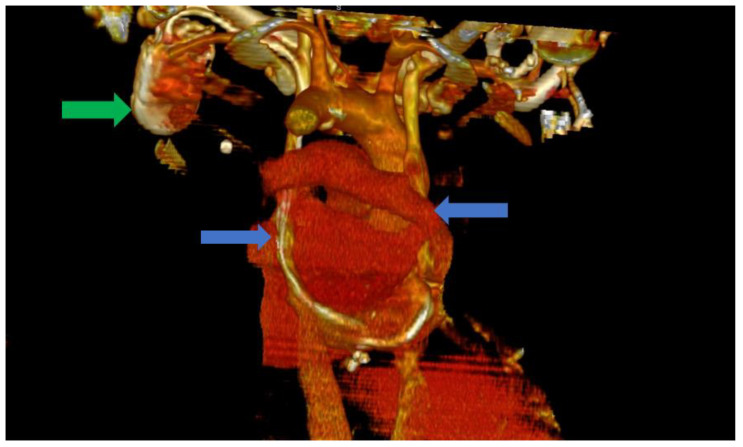
Computer tomography of the heart ang large vessels; in this scenario, a posterior view is provided. We can clearly observe the two superior vena cava (blue arrows) that do not have any communicating branches. In addition, the impulse generator and the adjacent leads can be observed (green arrow).

**Figure 4:**
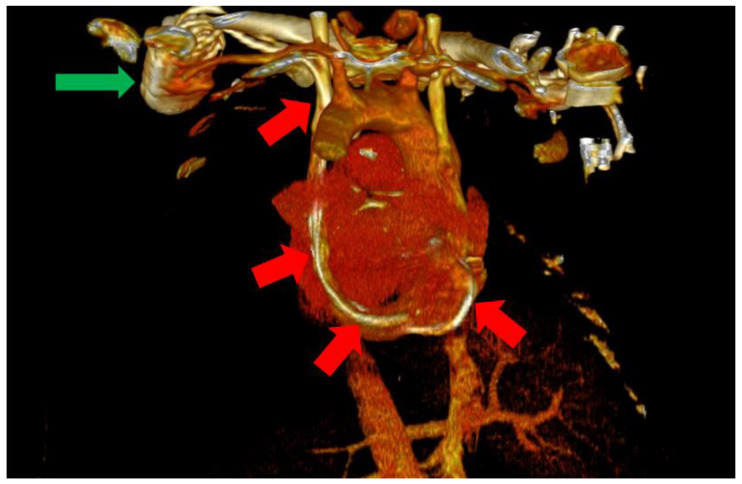
Posterior view of the heart and large vessels with most of the skeletal structure and soft tissue removed. The impulse generator (green arrow) and the leads can be clearly observed through the PLSVC (red arrows).

In this situation, we highly suspected a venous anomaly and the most probable anomaly was PLSVC. The patient was a clear candidate for CRT-D, with a class IIA indication according to the current guidelines [1]. Thus, the following step was the implantation of a biventricular ICD (St. Jude Medical Quadra Assura). When it came to choosing between CRT-D and CRT-P, we took several factors into consideration, including our clinical experience with both types of devices, as well as the patient’s age. Several studies in the literature, including trials and meta analyses, support the benefits of CRT-D over CRT-P, with some studies showing significantly lower all-cause mortality in patients who also benefit from the defibrillation function of the device and are aged under 75 years old [8,9]. We consider, from our clinical experience, that the additional protection offered by a CRT-D device is of great value in these patients, offering significantly more protection. The atrial lead was excluded as the patient was considered to be in permanent atrial fibrillation (AFib) with little or no chances of a long-term conversion to sinus rhythm. The size of the atria (a left atrial volume of 190 mL) and the grade III mitral regurgitation were strong arguments in this regard. The left subclavian vein approach was used in order to comply with the patient’s needs. Regarding the possibility of AFib ablation and atrioventricular (AV) node junction ablation, there are several factors that must be discussed. The atria of our patient were also significantly dilated, with the aforementioned dimensions and the patient had been in AFib for approximately a year. Thus, after taking these aspects into consideration, as well as discussing the benefits and risks of AFib ablation, we decided against AFib ablation or electrical cardioversion, since the real-life chances of success and the hopes of maintaining long-term sinus rhythm were low, guiding ourselves by the aforementioned anatomical modifications, as well as the period of time in which the patient had been in sustained AFib. In terms of atrioventricular node ablation, there are studies that have shown its superiority over conventional pharmacological treatment [10]. In our particular situation, we managed to easily obtain rate control via pharmacological methods and decided, together with the patient, against the procedure, again taking into consideration the highly unusual anatomy. Since the computer tomography angiography (angioCT) for an appropriate thoracic venous system anatomy description was not available before the procedure, the diagnosis of PLSVC was confirmed during the intervention by contrast venography and by the trajectory of the guidewires and leads. This required the introduction of the right ventricular (RV) lead (St. Jude Medical Durata 7122Q 58 cm) using the alfa loop technique, a method that consists of using multiple curved stillets in order to achieve the necessary angle and curvature to properly position the defibrillation lead within the RV, achieving a pacing threshold of 1 V and a sensing value of around 10 mV. The appropriate positioning was obtained with significant difficulty. Regarding the coronary sinus (CS) lead, a St. Jude Medical Quartet 1458Q was chosen due to the device supporting a quadripolar lead configuration, giving the physician more options in terms of depolarization vectors. The identification of a posterior coronary sinus branch proved to be extremely difficult, due to the sheer size and volume of the coronary sinus, which made the positioning of the quadripolar passive fixation CS lead nearly impossible. After several attempts, a satisfying position was achieved with adequate pacing and sensing parameters, with a minimum pacing threshold of 2.5 V on all electrodes and a sensing value of around 8.5 mV. However, in the following days after the procedure, the surface ECG revealed QRS complexes with aspects suggestive of right ventricular pacing instead of biventricular pacing. The postero-anterior chest X-ray raised the suspicion of a migrated CS lead. Within the following days, a thoracic angioCT was performed, which clearly showed the presence of PLSVC draining into the coronary sinus, with an equal size to the right vena cava, and without demonstrating any significant communicating branches (Figure 3 and Figure 4).

**Figure 5:**
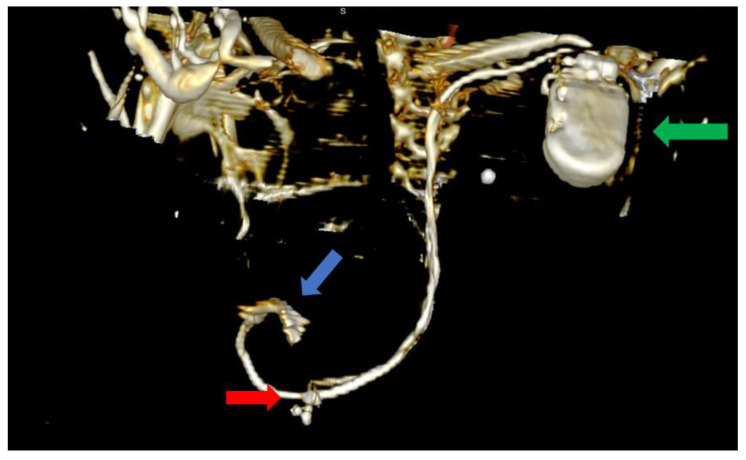
Computer tomography showing an anterior view with the soft tissue removed from the image, as well as most of the skeleton. The image clearly shows the impulse generator (green arrow), the right ventricular lead implanted in the right ventricle forming a loop (blue arrow), as well as the tetrapolar CS lead adjacent to the right ventricle; the lead had migrated from its initial position in the CS (red arrow).

**Figure 6:**
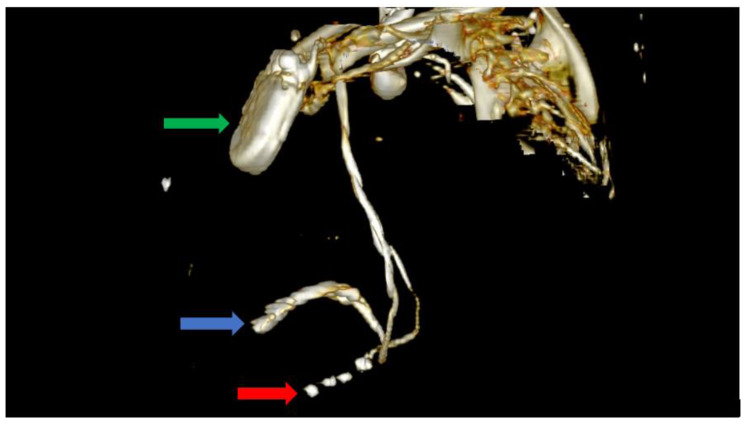
Computer tomography showing a left lateral view with the soft tissue removed from the image, as well as most of the skeleton. The image clearly shows the impulse generator (green arrow), the right ventricular lead implanted in the right ventricle forming a loop (blue arrow), as well as the tetrapolar CS lead adjacent to the right ventricle; the lead had migrated from its initial position in the CS (red arrow).

Furthermore, we could observe the migrated CS lead, which was now positioned adjacent to the RV and was capable of delivering RV pacing. The defibrillation lead remained in its proper position near the apex of the RV where it had been positioned using the alfa-loop technique (Figure 5 and Figure 6).

In order to avoid a second intervention for CS lead repositioning with its associated risks, we tried to adjust the device’s parameters, taking advantage of the benefits of the quadripolar CS lead. While we were able to identify adequate pacing and sensing thresholds, we were not able to obtain true biventricular pacing with the CS lead in this position (Figure 5 and Figure 6).

## 3. Discussion and Literature Review

PLSVC without an innominate vein generally does not raise any concerns, with individuals usually living normal lives without the anomaly being discovered [7], making its prevalence in the general population difficult to evaluate. However, in patients requiring intracardiac devices, the difficulty of the procedure increases significantly. While the procedure of pacemaker implantation in patients requiring permanent electrostimulation faces only the problem of venous access to the right cardiac chambers (i.e., right atrium and left ventricle), placing a lead within the CS complicates the situation drastically.

CRT therapy is an established form of treatment in patients such as the one presented in our manuscript and will probably remain so in the near future. Several advancements such as His bundle pacing optimized CRT have been made in recent years. While these techniques show promise and may pave the way towards obtaining physiological-like QRS complexes in patients with LBBB, several studies have been conducted and have not yielded definitive results regarding the superiority of His bundle optimized therapy over conventional CRT [11,12]. While this was theoretically an option when discussing our case, it would have been very difficult to implement in our particular situation due to several reasons. One main reason would be the heavily modified venous and cardiac anatomy. Another paramount reason would be the concerns regarding the stability of the RV defibrillator lead, more precisely the risk of dislodgement compared to a relatively more traditional apical position. Since the defibrillation lead had to be positioned in the right ventricle using the alpha loop technique, even in theory, it would have been much more difficult to obtain a stable position in order to achieve His bundle pacing or left bundle branch pacing. There have been reports of significant lead-related complications necessitating lead revisions even in patients with common venous and heart anatomies [13]. With these aspects in mind, we opted for what we considered to be the most stable position for the RV lead due to the fact that we consider it to be the “life-saving lead” in case malignant ventricular arrhythmias occur.

Two factors made positioning of the CS lead difficult. The first was the presence of the PLSVC, which, as stated earlier, drained into the CS, leading to an extremely modified venous drainage system. The second factor was the presence of DCM, which meant we had to deal with a very modified heart structure along with the venous system. While similar procedures have been reported, these were carried out on patients with higher ejection fractions and lower ventricular volumes. Despite the similarity to physiological anatomy, the authors reported difficulties cannulating due to the amount of blood flow through the modified CS. In this case, even with the use of selective catheters and support wires, cannulation proved extremely difficult [14]. While placement within the CS was achieved, these positions were suboptimal when compared to the CS lead locations in patients without PLSVC. Other authors considered using a pentapolar right ventricular lead and a VDD pacing system and reported CRT therapy being achieved by using only the proximal electrode of the CS lead. However, the presented patient had far smaller ventricular volumes and greater ejection fraction with an anatomy closer to physiological values in comparison to our case [15]. While our clinic also has experience using VDD devices [16], our patient was considered to be in permanent AFib; thus, an atrial lead or VDD system was not taken into consideration.

With these aspects in mind, our results attempting to use a passive fixation, 86 cm quadripolar CS lead (St. Jude Medical Quartet 1458Q) did not yield a completely successful outcome when facing such a modified and dilated venous system, along with a very far from normal heart anatomy. Even though we used a smaller angiography catheter within the CS CRT delivery system, the procedure lasted for over 2 h, as it was extremely difficult to locate any branch of the CS. While there are few cases reported in the literature, some authors have reported successfully achieving CRT by means of passive fixation CS leads by approaching via the left venous system [17]. In this case, satisfactory electrical results were achieved by means of QRS narrowing, but the authors provided no details regarding heart volume or ejection fraction. Another study reported achieving successful CRT criteria by using an active fixation CS lead in a patient with similar ejection fraction, although the study did not provide detailed volumetric echocardiographic details [18].

Some authors whom have encountered similar situations, albeit with defibrillators and not CRT-D, have suggested continuing via the left subclavian route if PLSVC is detected during the procedure and approaching via the right subclavian vein if the left-sided approach fails [19]. In our case, there was a high suspicion of PLSVC before the procedure. However, after discussing the choice between the left and right subclavian approach before the intervention with the patient and taking his option for a left-sided device into consideration, as well as taking our clinical expertise into consideration, having encountered similar cases before, we considered left subclavian access to be feasible. Thus, we decided on this approach, bearing in mind the potential difficulties but also considering the fact that there were chances of success via the “classical” route.

Our concerns regarding heavily modified anatomies encountered while attempting the implantation of a CRT-D device can be backed up by a postmortem analysis of a patient with LSVC, which has also demonstrated the heavily modified anatomy of the venous system of the heart, noting abnormalities such as the absence of the CS ostium valve, alongside other modifications to the heart’s anatomy [4]. Overall, the case we presented is quite special not only due to the presence of the LSVC, which was suspected by simple transthoracic ultrasonography, but also due to the altered anatomy of the heart in the context of dilated cardiomyopathy.

## 4. Conclusions

Patients with echocardiographic signs of PLSVC and DCM that require the implantation of a CRT-D device are a rare occurrence and pose a significant challenge for the interventionist, especially when using the classic left subclavian vein approach. However, steps can be taken and preparations can be made in order to ensure successful device implantation, as well as obtaining adequate biventricular pacing. Firstly, these patients should be investigated via vascular thoracic CT in order to visualize the nature of the malformation and observe the presence or absence of an innominate vein, as this has a direct impact on the course of the procedure. Furthermore, proper active fixation leads should be used in order to avoid lead dislodgment. Keeping our experience in mind, while there are cases reported in the literature in which CRT-D implantation has been successful via the left subclavian route, if CRT therapy fails when approaching from the left side, a right-sided attempt is a valid option if the patient is comfortable with the device in this respective position. Approaching via the right subclavian route can also be taken into consideration as a first line option if PLSVC is suspected before the procedure through imagistic means and if the patient has highly modified cardiac anatomy, as both of these elements may decrease the chances of a successful left-sided approach. Finally, the patients should be compliant not only during the procedure, as operation times will most definitely be prolonged due to the anatomical challenges and the risk of complications, especially lead dislodgment, are significantly higher when compared to patients with ordinary venous anatomy. Patients must also attend routine check-ups, as they are important in order to obtain long-term biventricular pacing and ensure no complications have taken place.

## Data Availability

Data are contained within the article.
